# Nitrosative Stress Biomarkers in the Non-Stimulated and Stimulated Saliva, as well as Gingival Crevicular Fluid of Patients with Periodontitis: Review and Clinical Study

**DOI:** 10.3390/antiox9030259

**Published:** 2020-03-21

**Authors:** Joanna Toczewska, Tomasz Konopka, Anna Zalewska, Mateusz Maciejczyk

**Affiliations:** 1 Department of Periodontology, Wrocław Medical University, 50-425 Wroclaw, Poland; tomasz.konopka@umed.wroc.pl; 2 Experimental Dentistry Laboratory, Medical University of Bialystok, 15-222 Bialystok, Poland; azalewska426@gmail.com; 3 Department of Hygiene, Epidemiology and Ergonomics, Medical University of Bialystok, 15-222 Bialystok, Poland; mat.maciejczyk@gmail.com

**Keywords:** periodontal disease, nitrosative stress, saliva, gingival crevicular fluid, salivary diagnostics

## Abstract

Diagnosis of periodontopathy is complex and includes defining the cause, type, stage, and grade of periodontitis. Therefore, alternative diagnostic methods are sought to indicate the progression of inflammation or to determine the effectiveness of therapy. Gingival crevicular fluid (GCF) biomarkers can be particularly useful because they most likely reflect the disease process of the periodontal tissues. However, the difficulty of collecting GCF for testing is the reason for the limited use in diagnostics. Because periodontitis is the primary source of nitrogen free radicals in the oral cavity, the aim of the study was to evaluate the biomarkers of nitrosative stress (nitric oxide, peroxynitrite, and *S*-nitrosothiols) in GCF, non-stimulated and stimulated saliva of 90 patients with periodontitis. The study group was divided into two subgroups, depending on the stage of the disease severity. We showed a significantly higher concentration of all assessed biomarkers in the non-stimulated and stimulated saliva of patients with periodontitis. However, significant changes in GCF has been shown only for peroxynitrite. The studied biomarkers did not correlate with clinical periodontal status, which probably results from their short-duration activity and the impact on a few factors in the oral cavity. Saliva and gingival fluid are not very useful in the differential diagnosis of periodontitis.

## 1. Introduction

The most recent definition of periodontitis indicates that it is a chronic and multifactorial inflammatory disease associated with dysbiosis in bacterial biofilm and leading to progressive destruction of the attachment apparatus [1]. The lesions associated with periodontitis can be tracked at the level of periodontal pathogens of the biofilm in the periodontal pocket (microbiome), markers of an immune-inflammatory response in the connective tissue of the gingiva, peridentium and cells, as well as mediators of alveolar bone remodeling and expression of multiple genes (genome and epigenome). The picture of periodontopathy is also influenced by the effects of the immune-inflammatory response, contained in the gingival crevicular fluid (GCF) and saliva, as well as the impact of environmental and behavioral factors (proteome, metabolome, transcriptome).

Evidence has shown that interaction of phagocytic cells with invading pathogens results in the nitric oxide (NO) formation (primarily form inducible nitric oxide synthases—iNOS) and superoxide anion, leading to the formation of peroxynitrite, responsible for the destruction of microorganisms [2]. Products of cell-wall degradation of bacteria and peroxynitrite trigger a positive feedback loop, which results in further iNOS induction and excessive production of NO and its derivatives [3]. Since NO has a limited ability to react with cellular elements, most of the deleterious effects associated with NO overproduction are attributed to peroxynitrite or other derivatives of nitric oxide. It should be emphasized that the excessive production of NO derivatives may lead to the cytotoxicity towards the host’s own tissues, which results in their destruction through reactions such as oxidation and nitrosylation, inhibition of mitochondrial enzymes, and DNA changes. Indeed, Ozmeric et al. proved that a bacterial lipopolysaccharide of periopathogenic bacteria leads to apoptosis of periodontal ligament cells by burst iNOS activity, followed by the localized microvasculopathy, ischemia, and permanent damage to endothelial and periodontal tissue [4]. Furthermore, research by Wang et al. has shown that NO has a role in the occurrence and development of periodontal disease by upregulation of proinflammatory cytokine activity [5].

Parwani et al, Aurer et al., and Ozmeric et al. have demonstrated that salivary NO levels in patients suffering from gingivitis and periodontitis were higher than in the healthy group [4,6,7]. Furthermore, the intensification of the inflammatory process, in the face of the shift to a more destructive stage, has resulted in increasing NO levels; therefore, the authors have concluded that salivary NO levels may be a determinant of the patients’ inflammatory status.

As part of the World Workshop on the Classification of Periodontal and Peri-implant Diseases and Conditions, a new system of classification of periodontal diseases was developed. Diagnosis for periodontopathy includes: defining a case as periodontitis, identifying a form of periodontitis, and describing a stage and a grade of periodontitis. Stages describe the severity of disease at the time of treatment and the treatment plan complexity. Grades provide information about the disease characteristics, a rate of its progression, a risk assessment, an analysis of possible treatment failures, an impact assessment of periodontopathy, and its treatment as regards the patient’s general condition [8,9]. Although clinical diagnostics of periodontitis is a widely used and proven method, it is also time consuming as well as it detects changes at a later stage of the disease. Currently, biochemical parameters are being sought to predict the rate of disease progression, which would lead to planning a more accurate dental treatment. However, among all of the salivary biomarkers, it is the oxidative/nitrosative stress products that are becoming increasingly popular [10,11,12,13].

Given the critical role of redox imbalance in the pathogenesis of oral inflammation, the aim of this research is to evaluate the selected biomarkers of nitrosative stress (nitric oxide, peroxynitrite, and S-nitrosothiols) in relation to the stages of periodontitis according to the new classification of periodontal diseases [8,9]. The study also includes the interchangeabilities between the oxidative stress parameters and clinical markers of periodontitis.

## 2. Materials and Methods 

### 2.1. Patients

The study has been approved by the Bioethics Committee of the Medical University named after Piastów Śląskich in Wrocław (KB-559/2018). The study was conducted in the period from 19 February 2018 to 31 March 2019. 

The research involved 60 patients treated for periodontitis in the Department of Periodontology at the Medical University named after Piastów Śląskich in Wrocław. The diagnosis was established on the basis of a clinical examination, in accordance with the currently accepted definition of periodontitis [8]. The patients were Caucasian Poles, aged 20–55.

The study group was divided into two subgroups, depending on the stage of disease severity, in accordance with the guidelines established during the World Workshop on the Classification of Periodontal and Peri-implant Diseases and Conditions [9]—Stage III (36 subjects) and Stage IV (24 subjects). 

The control group (C) involved 30 persons with clinically healthy periodontium (BOP (bleeding on probing) < 10%, PD (pocket depth) ≤ 3 mm), matched with age and gender to the study group, who had been admitted to treatment at the Academic Dental Clinic.

The exclusion criteria for the study and control groups were: age under 20 and over 55 years, coexisting systemic diseases related to oxidative stress (cancer, hypertension, diabetes, insulin resistance, obesity, rheumatoid arthritis, kidney, lung, liver, and thyroid diseases), pregnancy, the use of medicines and dietary supplements for 3 months before the trial, current nicotinism and alcoholism, a number of teeth less than 15, the occurrence of pathological lesions on the oral mucosa, and periodontal treatment less than a year before the trial. All individuals did not practice increased physical activity for 24 h prior to the study.

### 2.2. Sample Collection

After being classified to the study or control group, mixed saliva (both stimulated and non-stimulated) and gingival crevicular fluid (GCF) were collected from the patients. 

Saliva collection took place between 8 and 10 a.m., in a separate room to avoid lack of comfort and nervousness of the patient. Saliva was collected in a sitting position and after a 5 min adaptation period, with the head slightly inclined downwards and minimizing facial and labial movements. Saliva was collected using the spitting method. The saliva accumulated at the base of the oral cavity was spat into a sterile Falcon^®^ test tube. After rinsing the mouth with room-temperature water three times, the saliva was collected in a sterile Falcon^®^ test tube which was then placed in an ice container. Non-stimulated saliva was spit in to a maximum volume of 5 mL, within no more than 10 min [13]. Stimulated saliva was collected for 5 min. The secretion of saliva was stimulated by administering 10 µL of 2% citric acid on the tongue every 30 seconds [14]. The volume of saliva was measured with an automated pipette, with an accuracy of 0.1 ml. Immediately after collecting, samples were centrifuged (5000× *g*, 20 min, 4 °C). The supernatant fluid was preserved for the study, to which an antioxidant (10 µL of 0.5 M butylated hydroxytoluene per 1 mL of saliva) was added [15]. Such samples were frozen at −80 °C. 

A salivary flow rate was calculated by dividing the volume of saliva by the time necessary for its secretion and expressed in ml/min [16].

The gingival crevicular fluid was collected from the clinically deepest periodontal pockets using PerioPaper Strips^®^. The region was isolated from saliva access by means of cotton dental rolls and then it was dried with a compressed air. Before and after collecting the material, PerioPaper Strips^®^ were placed in Eppendorf^®^ test tubes and weighed on an analytical balance to determine the volume of gingival crevicular fluid. The samples were frozen at −80 °C. The PerioPaper Strips^®^ contaminated with blood or saliva were discarded.

### 2.3. Clinical Examination

The clinical condition of periodontium was assessed on the basis of a periodontal clinical trial, in accordance with the original chart developed for the study. Tooth mobility was assessed using Periotest^®^ (maximum and average value from the measurements made). The clinical trial was carried out using artificial lighting, a dentist’s mirror, and a periodontometer calibrated every 1 mm. The following variables were evaluated: modified PI (plaque index) by O’Leary et al. [9], assessing, as a percentage, the occurrence of the supragingival plaque on vestibular and lingual surfaces of the teeth. API (approximal plaque index) by Lange et al. [17], assessing, as a percentage, the occurrence of the plaque in interdental spaces. Bleeding on Probing (BoP) index by Ainamo and Baya [18], examined on 6 regions of each tooth: proximal buccal, median buccal, distal buccal, proximal lingual, median lingual, distal lingual. Papillary Bleeding Index (PBI) by Saxer and Mühlemann [19], assessing the intensity of periodontal inflammation. The pocket depth (PD) on 6 regions of each tooth: proximal buccal, median buccal, distal buccal, proximal lingual, median lingual, distal lingual. Clinical attachment level (CAL) on 6 regions of each tooth: proximal buccal, median buccal, distal buccal, proximal lingual, median lingual, distal lingual. Average PD for all teeth, measured on 6 regions of each one, and average interproximal PD for all teeth, measured on 4 regions of each tooth: proximal buccal, distal buccal, proximal lingual, distal lingual (number of regions with PD > 5 mm). A percentage of teeth with CAL (clinical attachment level) ≥ 5 mm on interproximal surfaces (on 4 regions of each tooth: proximal buccal, distal buccal, proximal lingual, distal lingual), a percentage of regions with CAL > 0 mm, and average CAL on interproximal surfaces with CAL regions > 3 mm. The clinical trial was conducted by one calibrated researcher.

### 2.4. Redox Assays

On the day of determining, the samples containing saliva and gingival crevicular fluid were slowly thawed at 4 °C. In order to extract gingival crevicular fluid, PerioPaper Strips^®^ were placed in an Eppendorf^®^ test tube containing 0.02 M phosphate-buffered saline solution (PBS) with pH of 7.0 (1 PerioPaper Strips^®^/500 µL PBS). Samples were mixed for 30 s with a vortex mixer and then centrifuged (4 °C, 3000× *g*, 5 min) [20]. The supernatant fluid was preserved for testing. An antioxidant (10 µL of 0.5 M butylated hydroxytoluene per 1 mL of gingival crevicular fluid) was added to the samples containing GCF and mixed with a vortex mixer [15]. The gingival crevicular fluid was used for all markings on the same day. All samples were mixed immediately prior to determining.

The levels of total protein were determined colorimetrically by means of the commercial Thermo Scientific PIERCE BCA Protein Assay set (Rockford, IL, USA) using the bicinchoninate method. The absorbance of samples was measured at 562 nm and the levels of total protein were read from the standard curve for bovine serum albumin (BSA). The levels of total protein were expressed in µg/mL.

Nitric oxide (NO) levels were determined by colorimetric analysis, described by Grisham et al. [21]. The principle of this method is based on the nitrate reaction (3+) with sulphanilamide and *N*-(1-naphthyl)-ethylenediamine dihydrochloride, which form a color product with a maximum absorption wavelength of 490 nm. To determine the NO levels, 100 µL of the sample was incubated from 100 µL of freshly prepared Griess reagent (1% of sulphanilamide solution and 0.1% of *N*-(1-naphthyl)-ethylenediamine dihydrochloride in 2.5% metaphosphoric acid. After 15 min of dark incubation, the absorbance of samples was measured at (a wavelength of) 490 nm. Nitric oxide levels were calculated from the standard curve for sodium nitrate (3^+^). The NO levels were determined in double assays and expressed in µM/mg of total protein.

The levels of *S*-nitrosothiols were measured by the colorimetric analysis, described by Grisham et al. [22]. The principle of this method is based on the reaction of Griess reagent with *S*-nitrosothiols contained in the test sample, followed by the reaction with Hg^2+^ mercury ions. The maximum absorption of the created complex occurs at 490 nm. To determine the levels of *S*-nitrosothiols, the sample was incubated with the freshly prepared Griess reagent and with 10 mM of mercuric chloride solution (2^+^). The reaction-mixture was thoroughly mixed and after 20 min of dark incubation at room temperature, the absorbance of the created complex was measured at 490 nm. The molar absorption coefficient (ε = 11,500 M^−1^cm^−1^) was used to calculate the levels of *S*-nitrosothiols. The levels of *S*-nitrosothiols were determined in double assays and expressed in µM/mg of total protein.

The peroxynitrite levels were determined by fluorimetric method, measuring the degree of nitrosylation of phenol. The *S*-nitrophenol, as the result of a reaction between peroxynitrite and phenol, shows maximum absorption at an excitation wavelength of 490 nm and at an emission wavelength of 530 nm. For the determination of peroxynitrite levels, 50 µL of the assay was incubated with the same volume of reaction-mixture, which was diluted 500 times with 0.6 M sodium nitrate, 0.7 M hydrogen peroxide in the medium of 0.6 M hydrochloric acid, and 1.5 M sodium peroxide. The assays were incubated for 10 min in the dark at room temperature. Fluorescence-based assays were measured at an excitation wavelength of 490 nm and at an emission wavelength of 530 nm. The molar absorption coefficient (ε = 1670 M^−1^cm^−1^) was used to calculate the levels of peroxynitrite. The peroxynitrite levels were determined in double assays and expressed in µM/mg of total protein. 

### 2.5. Statistical Analysis

In a statistical analysis, due to lack of normal distribution of all variables (which was verified with the Shapiro–Wilk test), the following tests were used: the Mann–Whitney U test to compare two groups, the analysis of variance (ANOVA) Kruskal–Wallis test—for three groups, and the Dunn’s post-hoc test. Multiplicity adjusted *p* value was also calculated. The Spearman’s test was used in the analysis of the interchangeability between two variables. The assumed threshold for statistical significance was *p* ≤ 0.05, whereas in the correlation analysis, it was *p* ≤ 0.02. The analysis was conducted using the statistical package: Statistica 13.1 (StatSoft, Wrocław, Poland).

The number of patients was determined based on our previous pilot study. The power of the test was assumed to be 0.9.

## 3. Results

General and periodontal data of patients are presented in Table 1.

### 3.1. Nitric Oxide (NO)

**Figure 1:**
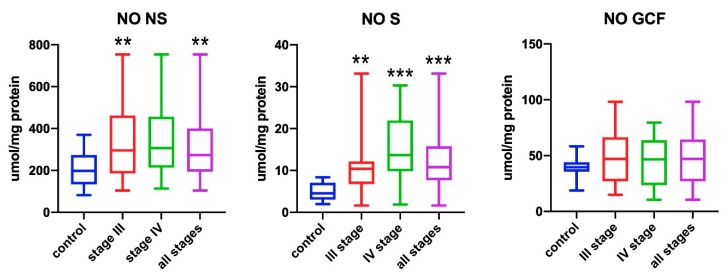
Concentrations of nitric oxide (NO) in non-stimulated saliva (NS), stimulated saliva (S), and gingival crevicular fluid (GCF) in all patient groups. ** *p* < 0.01 *** *p* < 0.001 versus control group.

The nitric oxide levels in non-stimulated saliva in patients suffering from periodontitis were significantly higher than in healthy subjects (*p* = 0.006). The patients with stage III periodontitis had significantly higher levels of nitric oxide in comparison to healthy subjects (*p* = 0.001) (Figure 1). 

The levels of nitric oxide in stimulated saliva of all patients were significantly higher in comparison to healthy subjects (*p* < 0.001). Significantly higher levels of nitric oxide were observed in stage III and IV patients than in the control group (*p* = 0.002 and *p* < 0.001, respectively). Patients with stage IV periodontitis showed higher levels of nitric oxide than stage II and III patients (*p* < 0.001) (Figure 1).

No statistically significant differences in the nitric oxide levels in GCF were found between the studied groups (Figure 1).

### 3.2. Peroxynitrite

**Figure 2:**
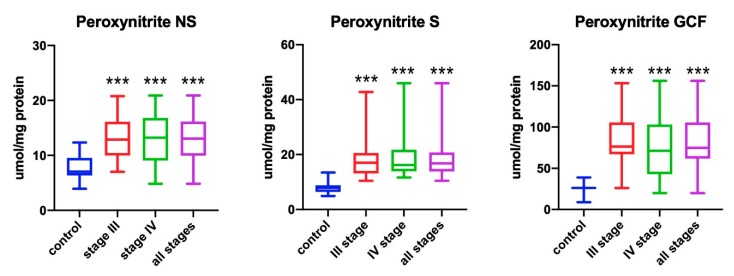
Concentrations of peroxynitrite in non-stimulated saliva (NS), stimulated saliva (S), and gingival crevicular fluid (GCF) in all patient groups. *** *p* < 0.001 versus control group.

The peroxynitrite levels in non-stimulated saliva of all patients was significantly higher than in healthy subjects (*p* < 0.001). The patients with stage III and IV periodontitis had significantly higher peroxynitrite levels in comparison to healthy subjects (*p* < 0.001 and *p* < 0.001, respectively) (Figure 2).

The peroxynitrite levels in stimulated saliva of all patients were significantly higher in comparison to healthy subjects (*p* < 0.001). Stage III patients showed significantly higher concentration than the control group (*p* < 0.001), which was also confirmed for stage IV patients (*p* < 0.001) (Figure 2). 

The peroxynitrite levels in GCF of all patients were significantly higher in comparison to healthy subjects (*p* < 0.001). Significantly higher concentration was found in stage III and IV patients in comparison to the control group (*p* < 0.001 for stage III and *p* < 0.001 for stage IV, respectively) (Figure 2).

### 3.3. S-Nitrosothiols

**Figure 3:**
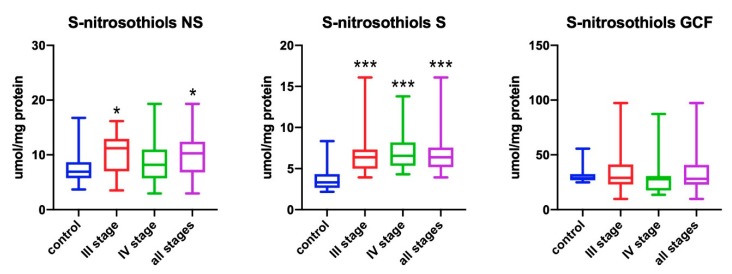
Concentrations of *S*-nitrosothiols in non-stimulated saliva (NS), stimulated saliva (S), and gingival crevicular fluid (GCF) in all patient groups. * *p* < 0.05, *** *p* < 0.001 versus control group.

The levels of *S*-nitrosothiols in non-stimulated saliva of all patients were significantly higher in comparison to healthy subjects (*p* = 0.0219). The stage III patients had significantly higher levels of *S*-nitrosothiols in comparison to healthy subjects (*p* = 0.023) (Figure 3). 

The levels of *S*-nitrosothiols in stimulated saliva of all patients were significantly higher in comparison to healthy subjects (*p* < 0.001). Significantly higher concentration was found in stage III and IV patients in comparison to the control group (*p* < 0.001 for stage III and *p* < 0.001 for stage IV, respectively) (Figure 3).

No statistically significant differences in the levels of S-nitrosothiols in GCF were found between the studied groups (Figure 3).

### 3.4. Correlations

The analysis of correlation between nitric oxide levels in non-stimulated saliva of the whole group of patients, as well as stage IV of the disease, and clinical markers of the disease, did not show any significant interchangeability. The analysis of correlation between nitric oxide levels in stimulated saliva of the whole Perio group, as well as stage IV of the disease, and clinical parameters, showed only a significant positive correlation for stage IV with the genetic load—periodontitis, confirmed by medical history (R = 0.6; *p* = 0.01). Likewise, the only significant correlation regarding nitric oxide levels in GCF was observed for stage IV with average CAL on interproximal surfaces with CAL regions ≥ 3 mm (R = 0.72; *p* = 0.002). 

In the analysis of correlation regarding peroxynitrite levels in the three biological fluids studied, the significant interchangeability has been found between GCF concentrations in stage IV of the disease and genetic load—periodontitis, confirmed by medical history (Rv = 0.59; *p* = 0.015). The levels of *S*-nitrosothiols in GCF and saliva did not correlate significantly with the markers of clinical conditions of periodontium. 

### 3.5. GCF Biomarkers

**Figure 4:**
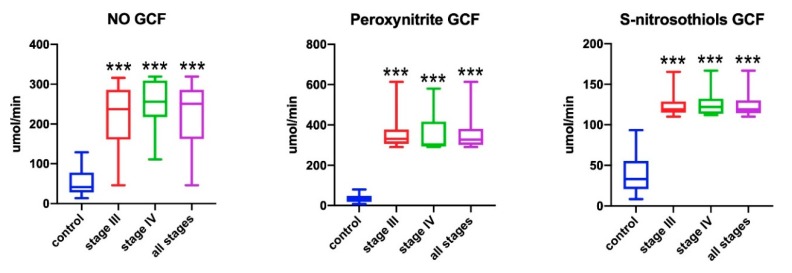
Concentrations of nitric oxide (NO), peroxynitrite, and *S*-nitrosothiols in gingival crevicular fluid (GCF) in all patient groups. *** *p* < 0.001 versus control group.

In the course of periodontopathy, increased protein secretion to GCF occurs. Therefore, nitrosative stress biomarkers can be standardized to the time of GCF collection [23]. After standardization, the concentration of NO, peroxynitrite, and *S*-nitrosothiols was significantly higher in GCF of patients with periodontitis compared to controls (*p* < 0.001) (Figure 4). However, analyzed GCF biomarkers did not differentiate stage III and IV periodontitis. We also did not observe any statistically significant correlations between GCF biomarkers and periodontium clinical condition.

## 4. Discussion

This is the first study comparing nitrosative stress biomarkers in the non-stimulated and stimulated saliva as well as gingival crevicular fluid in patients with periodontitis. We found no relationship between various bioliquids, as well as the severity of periodontitis.

Modern diagnosis for periodontopathy is based on findings of the World Workshop on the Classification of Periodontal and Peri-implant Diseases and Conditions and includes descriptive identification of a form of periodontitis, and description of a stage and a grade of periodontitis. The improvement of the previous classification is to lead to a flawless diagnosis for periodontitis and planning of an adequate treatment. Currently, biochemical methods, compatible with a new classification, are being sought.

Table 2 shows the test results on nitric oxide levels in GCF, saliva, and blood serum in periodontitis (the previous classification of periodontitis was used in tests) [6,7,24,25,26,27,28,29,30]. 

Despite the fact that the Griess reaction was the most frequently used method in the methodology for determining the nitric oxide, these test results are very heterogeneous. Significantly higher salivary NO levels in the course of periodontitis, as in our assessment, were obtained in five other studies [6,25,26,28,29], and significantly decreased in two others [7,27]. These differences resulted from many reasons. In the research by Aurera et al. [7], the exclusion criteria were not specified (e.g., active nicotinism), the control group involved dental students and a different method of saliva collection was used. In the study by Andrukhowa et al. [27], the mean serum values of NO and salivary nitrites in periodontitis were very similar (20 versus 8 mM), which was not confirmed by other authors (9.4 versus 79.5 µM of NO) [26], (20.05 versus 37.5 µM of NO) [30]. The serum NO levels are very susceptible to the methodology of saliva collection and storage. The nitric oxide per se is very nonpersistent. Furthermore, many salivary microorganisms reduce nitrates (V) to nitrates (III) at acidic pH. Obesity has also turned out to have a significant effect on the increase of salivary NO levels in periodontitis [31]. A meta-analysis by Chen et al. [32], involving three consistent studies (described in Table 2 [25,26,28]) and referring to 140 subjects suffering from periodontitis and 140 belonging to the control group, has shown a significant increase of NO levels in non-stimulated saliva in the course of periodontitis (*p* < 0.001, with a surprisingly high non-uniformity index of 93.4%). There is also a lack of full consistency between our assessment of NO levels in GCF in periodontitis (47.4 versus 40.8 µM, no significant difference) and significantly reduced concentration described by Poorsattar Bejeh-Mir (12.2 versus 32.1 µM, *p* = 0.019) [29]. It is possible to inhibit the NO secretion in GCF through the increased activity of arginase and ornithine decarboxylase (ODC) at the early stage of periodontitis [33]. The majority of studies indicate elevated blood serum NO levels in the course of periodontitis [24,25,26,28], although there are contradictory observations on this subject as well [27]. The majority of authors also observed the significant positive interchangeability between salivary [6,26,30] as well as serum [26,30] NO levels in periodontitis and clinical disease markers (poor oral hygiene, intensity of inflammation, as well as destructive lesions described by PD and CAL). In our study, only a significant positive interchangeability between NO levels in GCF in patients with the most severe stage of periodontitis and the decrease in clinical attachment level on interproximal surfaces has been indicated. Another interesting observation is the demonstration of a significant positive correlation between the NO levels in stimulated saliva in stage IV periodontitis and the family load of periodontitis (confirmed on the basis of medical history). This may indicate the role of genetic factors in NO secretion into saliva after stimulation. As it has been shown, in the Polish population, patients suffering from periodontitis carry the polymorphic rs2070744 genotype in the promoter section of the NOS3 gene located on chromosome 7q35–36 significantly more frequently [34]. 

In our study, the peroxynitrite levels were significantly higher in GCF for both types of saliva as well as in the course of both stages of periodontitis when compared to the control group. The peroxynitrite levels in GCF and both types of saliva did not differentiate stage III and IV periodontitis. A significant positive correlation between the peroxynitrite levels in GCF in stage IV periodontitis and a predisposition to familial periodontitis was observed as well. In the available literature, there is no reference to the evaluation of peroxynitrite in periodontopathy. The increase in the NO formation also confirms the role of nitrosative stress in the course of periodontitis, although no significant interchangeability between its concentration and clinical periodontal markers was found in two-factor analyses. Perhaps its activity under pH conditions of GCF, particularly saliva, is limited, but this requires further research. According to Topcu et al. [35], total nitrate (III) levels in GCF in patients suffering from chronic periodontitis were significantly higher in relation to the regions with clinically healthy periodontium, which was not shown for nitrates (V) in GCF and in saliva for both types of nitrogen metabolites. On that basis, a suggestion was made that nitrates (III) are more significant than nitrates (V) as potential biomarkers for periodontitis. Another study did not confirm those findings, as significantly higher salivary levels of nitrites and nitrates in periodontitis were described as regards the periodontally healthy control group, and there were no significant differences in nitrite levels in GCF between these groups [29]. Similarly, Mecharini et al. [36] did not indicate that the diagnosis for periodontitis significantly affected the levels of nitrites in blood plasma and non-stimulated saliva, whereas it was significantly decreased in stimulated saliva. The possible influence of a genetic component on the secretion of nitrates (III) and (V) in periodontitis is indicated by studies on the Brazilian population, which have shown that heterozygotes for two polymorphisms of the NOS2 -1026 (A > C) rs2779249 and +2087 (A > G) rs2297518 gene with CP secrete significantly less salivary nitrates (III) and (V) [37]. The levels of salivary nitrogen metabolites may also be affected by hormones, salivary secretion rate, and environmental exposures, e.g., to dietary components.

Reversible *S*-nitrosylation of cysteine or glutathione leads to the formation of *S*-nitrosothiols, which are signaling molecules and act as a nitric oxide reservoir and its transfer over long distances. In our assessment, the highest levels of *S*-nitrosothiols have been found in GCF in patients with stage III periodontitis; however, no difference in GCF levels in comparison to the control group has been shown. In both types of saliva, the levels of *S*-nitrosothiols were significantly higher in the study group, the difference in stimulated saliva was statistically significant for the most advanced stage of periodontopathy with regard to the control group. The levels of *S*-nitrosothiols in GCF and saliva did not correlate with periodontal markers either. The first known research in the available literature on the activity of *S*-nitrosylated proteins in periodontitis confirms their occurrence. In order to fully assess their biological effect in this pathological, it would be necessary to assess their concentrations in the vascular bed. 

In our study, we attempted to use saliva/GCF biomarkers of nitrosative stress in the diagnosis of periodontal disease. Salivary redox biomarkers are successfully used in the diagnostics of many systemic diseases such as diabetes, obesity, chronic kidney disease, chronic heart failure, neurodegenerative diseases, as well as cancer [11,12,13,16,38,39]. Indeed, the concentration of many biomarkers in saliva correlates with their content in the blood as well as disease progression. However, it should be remembered that the oral cavity is the only place in the body that is subjected to so many environmental factors [40]. These include: microorganisms (bacteria and viruses), food, xenobiotics (tobacco smoke, air pollutants, ethanol, and medicines), as well as dental treatment/materials. Most of them have been shown to have high oxidative potential and, therefore, can be a source of nitrosative stress in the oral cavity [40]. This indicates the main disadvantages of using saliva/GCF in biomedical diagnostics. As we showed here, saliva and gingival fluid are not very useful in the differential diagnosis of oral diseases such as periodontitis.

Although we have not demonstrated the diagnostic usefulness of the analyzed redox biomarkers, nitrosative stress plays an important role in the course of periodontitis. Therefore, antioxidant supplementation in patients with oral inflammation should be considered [41,42,43].

To summarize, our studies have confirmed an increase in salivary levels of nitrosative stress markers in the course of the most advanced stages of periodontitis. The studied nitrosative stress markers did not correlate significantly with the parameters of periodontium clinical condition, which probably results from their short-duration activity and the impact of the several external factors on the oral cavity.

## Figures and Tables

**Table 1:** General and periodontal data.

		Control					Stage III					Stage IV					All Stages				
		Mean	SD	Median	Min	Max	Mean	SD	Median	Min	Max	Mean	SD	Median	Min	Max	Mean	SD	Median	Min	Max
age		40.3	9.58	39	20	55	43.3	8.99	45	20	55	44	8.03	45	29	55	43.6	8.56	45	20	55
sex	women	17 (57%)					17 (47%)					13 (54%)					30 (50%)				
	men	13 (43%)					19 (53%)					11 (46%)					30 (50%)				
non-stimulated saliva flow (mL/min)		0.42	0.19	0.4	0.2	1	0.41	0.22	0.375	0.1	1	0.5	0.3	0.45	0.1	1.3	0.45	0.26	0.4	0.1	1.3
stimulated saliva flow (mL/min)		1.74	0.75	1.55	0.4	3.4	1.43	0.66	1.4	0.3	3	1.47	0.67	1.4	0.6	3	1.45	0.66	1.4	0.3	3
protein in non-stimulated saliva (µg/mL)		633.13	189.88	624.16	300.45	1101	865.87	237.05	824.55	481.23	1387	898.13	451.84	839.62	23.48	1847.1	879.15	338.4	827.95	23.48	1847.1
protein in stimulated saliva (µg/mL)		592.16	160.87	599.74	235.74	946.29	594.85	179.63	626.28	28.91	926.41	554.59	217.35	634.78	42.74	811.92	577.99	193.06	634.78	28.91	926.41
protein in gingival fluid (µg/mL)		39.23	21.56	31.22	8.44	91.66	132.30	69.83	130.43	36.8	336.97	178.78	109.71	134.24	45.5	445.64	151.82	90.75	131.13	36.8	445.64
number of teeth		26.1	2.63	27.5	19	28	26.86	1.53	27.5	21	28	22.21	4	23	15	28	25	3.59	26	15	28
PI		22.22	16.16	20.5	0	79	45.25	25.59	43	9	100	49.79	32.14	43.5	0	100	47.07	28.21	43.5	0	100
API		36.87	16.27	32	7	68	64.06	21.26	65	29	100	77.54	23.41	86.5	22	100	69.45	23.14	72.5	22	100
BoP		11.8	7.34	9.5	0.7	26	45.12	27.66	41	4	100	63.38	28.70	61	17	100	52.42	29.26	46.5	4	100
PD		1.74	0.32	1.7	1.2	2.3	3.19	0.66	3.15	2.1	5.3	4	0.61	4.1	2.7	5.4	3.51	0.75	3.5	2.1	5.4
mean CAL > 0		2.26	1.17	2.1	1	5.2	4.74	1.4	4.65	2.4	8.1	6.1	1.75	6.05	3	10.1	5.29	1.68	5.4	2.4	10.1

API—approximal plaque index; BoP—bleeding on probing; CAL—clinical attachment level; PD—probing depth; PI—plaque index; SD—standard deviation.

**Table 2:** Comparison of studies on nitric oxide (NO) concentration in periodontitis.

Author, Year and Country	Fluid Method	Study Group Size and Age	*p* for Perio	Other Data
Aurer et al. [7], 2001, Croatia	NS saliva S saliva Griess reaction	AgP, 25 (19–35) CP, 25 (39–59) HP, 25 (21–42)	NS saliva ↓ NO *p* < 0.001 S saliva ↓ NO *p* < 0.05	Decrease depends on periodontitis severity
Menaka et al. [24], 2009, India	Serum Griess reaction	CP, 30 (18–45) HP, 30 (18–45)	↑ NO *p* = 0.000	
Dhotre et al. [25], 2011, India	NS saliva Serum Cortas and Wakid method	P, 100 (mean 52.7) HP, 100 (mean 50.3)	Saliva ↑ NO *p* < 0.001 serum ↑ NO *p* < 0.001	
Parwani et al. [6], 2012, India	NS saliva Griess reaction	CP, 30 (20–60) HP, 30 (20–60)	↑ NO *p* = 0.000	Positive correlation with PD
Sundar et al. [26], 2013, India	NS saliva Serum Griess reaction	AgP, 20 (25–35) CP, 20 (35–55) HP, 20 (25–55)	Saliva ↑ NO *p* < 0.001 serum ↑ NO *p* < 0.001	Positive correlation for both with plaque, GI, PD, CAL, and concentration for saliva and serum
Andrukhov et al. [27], 2013, Austria	NS saliva (nitrite) Serum Griess reaction	Severe P, 89 (mean 34.3) HP, 54 (mean 42.2)	Saliva ↓ NO *p* < 0.01 serum ↓ NO *p* < 0.01	No correlation between saliva and serum
Wadhwa et al. [28], 2013, India	NS saliva Serum Griess reaction	CP, 20 (no data) HP, 20 (no data)	Saliva ↑ NO *p* < 0.05 serum ↑ NO *p* < 0.05	
Poorsattar Bejeh- Mir [29], 2014, Iran	GCF NS saliva ELISA	P, 14 (mean 38.3) HP, 14 (mean 37.7)	GCF ↓ NO *p* < 0.001 saliva ↑ NO *p* = 0.007	Sensitivity and specificity for NO in saliva in periodontitis is 0.93 and 0.96
Wattamwar et al. [30], 2016, India	NS saliva Serum Griess reaction	CP, 20 (30–55)	saliva ↑ NO *p* < 0.001 versus serum	Significant correlation for both with plaque, GI, PD, CAL, and between saliva and serum
Own study	GCF NS saliva S saliva Griess reaction	P, 60 (20–55) HP, 30 (20–55)	GCF, n.s. serum ↑ NO *p* < 0.001	In severe P in GCF positive correlation with interproximal CAL

NS saliva—non-stimulated saliva; S saliva—stimulated saliva; GCF—gingival crevicular fluid; P—periodontitis; CP—chronic periodontitis; AgP—aggressive periodontitis; HP—healthy patients; n.s.—statistically non-significant.
